# Editorial: Implementation science for disaster preparedness and emergency medicine

**DOI:** 10.3389/fpubh.2026.1950001

**Published:** 2026-08-28

**Authors:** Faraan O. Rahim, Sammer Marzouk, Lauren T. Southerland, Lauren Westafer, Julian T. Hertz

**Affiliations:** 1 Harvard Medical School, Boston, MA, United States; 2 Northwestern University Feinberg School of Medicine, Chicago, IL, United States; 3 The Ohio State University Wexner Medical Center, Columbus, OH, United States; 4 University of Massachusetts Chan Medical School–Baystate, Springfield, MA, United States; 5 Department of Emergency Medicine, Duke University School of Medicine, Durham, NC, United States; 6 Duke Global Health Institute, Durham, NC, United States

**Keywords:** disaster preparedness, emergency medical services, emergency medicine, implementation science, low- and middle-income countries

This Research Topic set out to advance implementation research in emergency care, with four aims: rigorous work on uptake of evidence-based practice in the emergency department (ED) and in prehospital and emergency medical services (EMS); new methods for implementation research in acute settings; de-implementation of low-value emergency testing and treatment; and attention to disaster preparedness and to resource-constrained settings, particularly low- and middle-income countries (LMICs), where disparities in access to evidence-based emergency care are widest. This Editorial reads the 13 contributions against those aims.

Implementation science studies the strategies, contexts, and behaviors that determine whether an evidence-based practice is adopted, delivered with fidelity, and sustained. Triage algorithms, evacuation plans, and outbreak responses all demand precise execution under pressure and across systems that vary enormously. Yet emergency and disaster medicine remain thinly represented in the formal implementation literature, which has concentrated on mental health, HIV, primary care, and chronic disease. A systematic scoping review of implementation research in emergency medicine found that most papers stopped at documenting evidence-practice gaps; only two used an explicit theoretical framework, and only five of 77 effectiveness studies used a randomized design (1, 2). Independent reviews of the West African Ebola epidemic and of COVID-19 both located the decisive failures in operations rather than science: delayed detection and alerting, fragmented coordination, and slow translation of existing guidance into field practice (3, 4).

Four questions run through the Research Topic: how hazards are communicated and managed across systems; how routine emergency and prehospital care builds the capabilities preparedness draws on; how the ED works as a platform for population health and continuity of care; and how equity shapes critical emergency outcomes. Table 1 gives each contribution with its country and income group, article type and design, and the preparedness capability it speaks to.

**Table 1:** Contributions to the Research Topic by theme, setting and World Bank income group, article type and study design, and preparedness-relevant capability.

Theme	Contribution (ref.)	Setting: country (World Bank income group)	Article type and study design	Preparedness-relevant capability
A. Hazard communication and cross-system management	Applying implementation science to infectious disease emergency preparedness and response (Øvretveit)	Sweden (high income)	Policy and practice review; conceptual guidance drawing on the author's account of Stockholm's COVID-19 response; no new data	Cross-agency coordination; structured iteration during evolving events
	Protocol for a community-engaged mixed-methods study for climate-related disaster preparedness and health care resilience (Tupetz et al.)	United States (high income)	Study protocol; mixed-methods surveys and focus groups across 15 counties; no results reported	Coalition building; mapping of partners, gaps, and resources
	Navigating the heat: heat alert communication and rural health data infrastructure (Rojo and Bosworth)	United States (high income)	Perspective; no primary data	Warning dissemination channels; surveillance and data systems
	Compound hazard risk index for natural and nuclear disasters, Aomori (Maekawa et al.)	Japan (high income)	Original research; vulnerability assessment of 108 medical institutions with damage-scenario overlay; support model proposed, not yet operating	Facility risk assessment; evacuation and surge routing
	Disaster knowledge, official trust, and warning sources in typhoon-prone areas (Wei et al.)	China (upper-middle income)	Original research; cross-sectional survey (*n* = 517) with structural equation modeling; outcome is risk perception, not protective behavior	Public trust and comprehension of warnings
B. Building routine emergency and prehospital capacity	Ultrasound-guided nerve block program in an academic ED (Brown et al.)	United States (high income)	Original research; completed implementation evaluation (PRISM and RE-AIM), 2021–2025	Workforce capacity; standing workflows, order sets, and supply chains
	Knowledge, attitudes, and practices of prehospital providers (Sah et al.)	Nepal (lower-middle income)	Original research; cross-sectional survey (*n* = 517) across 92 hub and satellite hospitals	Prehospital workforce capacity; knowledge-to-practice gap
	First aid knowledge among army soldiers (Krenzel and Narewska)	Poland (high income)	Original research; cross-sectional survey (*n* = 137)	Non-medical responder capacity assumed by surge plans
	Cost savings of EMS bronchodilators and corticosteroids for pediatric asthma (Fishe et al.)	United States (high income)	Brief research report; economic sub-analysis of stepped-wedge data from seven EMS agencies	Prehospital protocol standardization; conservation of inpatient beds
C. ED as a hub for population health and continuity of care	Deja Tu Huella ED-based HIV screening program (González del Castillo et al.)	Spain (high income)	Review; 4-year program report across 187 EDs	Multi-site testing and reporting infrastructure; linkage to care
	Telehealth fall prevention for older adult ED patients (Keleman et al.)	United States (high income)	Trial protocol; implementation feasibility as primary outcome; no results reported	Continuity of care after discharge; remote delivery
	ED-based educational intervention after acute myocardial infarction (Pyne et al.)	Tanzania (lower-middle income)	Original research; pre-post quasi-experimental study (*n* = 215)	Continuity of care beyond the emergency visit
D. Equity in critical emergency outcomes	Racial disparities, comorbidities, and low body mass index and survival after CPR (Du et al.)	Multiple countries (pooled); all race-stratified studies from the United States (high income)	Systematic review and meta-analysis of predominantly retrospective studies; associations, not causal effects	Equitable delivery of time-critical resuscitation

The largest group treats hazards broadly and converges on one point: communication and information flow are themselves implementation problems. A policy and practice review from Sweden offers the most explicit toolkit: how to distinguish interventions from implementation strategies, adapt to context, iterate in structured cycles, and coordinate across agencies. It draws on the author's account of Stockholm's COVID-19 response rather than on new data (Øvretveit). A study protocol from North Carolina aims to build a durable statewide coalition after Hurricane Helene, using surveys and focus groups across 15 affected counties; no results yet (Tupetz et al.). A perspective argues that email-based heat alerts and incomplete rural surveillance leave both the warning and the counting incomplete (Rojo and Bosworth). An original research article from Aomori, Japan develops a compound hazard risk index, applies it across 108 medical institutions, and overlays projected earthquake damage to identify facilities needing evacuation support; the prefecture-wide support model is proposed, not yet operating (Maekawa et al.). A cross-sectional survey of 517 residents of typhoon-prone Hainan, China found disaster knowledge and official trust positively associated with public risk perception, with the influence of warning sources fully mediated through them; the outcome was risk perception, not protective action (Wei et al.).

A second group reports on emergency and prehospital care. These are not disaster studies, and we do not read them as such. Their relevance is indirect but concrete: the capabilities a disaster consumes, ensuring an appropriately trained workforce, standing workflows and order sets, coordination across services, usable data, and continuity after the visit, are built in everyday operations and drawn down under surge. One academic ED took ultrasound-guided nerve blocks from zero to more than 50 procedures per quarter over 4 years by hiring a champion, building order panels with dosing safeguards, and embedding the technique in four clinical pathways; 93 residents and 34 faculty performed or supervised blocks, by building workforce capacity and standing workflows (Brown et al.). A survey of 517 Nepali prehospital providers evaluating the knowledge to practice gap found good knowledge in 62% but good practice in only 25%, locating the gap in workflow and supervision rather than training content (Sah et al.). A survey of 137 Polish soldiers found a similar knowledge to practice gap as roughly a quarter failed a first-aid test, with prior certification unrelated to score (Krenzel and Narewska). An economic sub-analysis of stepped-wedge data from seven EMS agencies estimated $6.9 million in averted pediatric asthma hospitalization costs nationally, rising to $26.4 million if targeted to mild exacerbations and long transports; hospitalizations averted in routine practice are also beds free for a surge (Fishe et al.).

Three contributions position the ED as a hub for population health and continuity of care. A review of Deja Tu Huella, an HIV screening program, reports 223,659 HIV serologies requested across 187 Spanish EDs in 4 years, yielding 2,357 new diagnoses and 87% linkage to care: a standing multi-site testing and reporting structure of the kind outbreak response requires (González del Castillo et al.). A trial protocol proposes adapting an ED-initiated fall-prevention intervention to telehealth for smaller EDs, with implementation feasibility as the primary outcome; no results yet (Keleman et al.). And furthermore, pre-post study in Moshi, Tanzania found higher 30-day use of antiplatelets (57% vs. 16%), beta-blockers (40% vs. 4%), and statins (42% vs. 8%) after an ED-based educational intervention following myocardial infarction, with no overall difference in disease understanding visit (Pyne et al.).

A systematic review and meta-analysis addresses equity. Pooling predominantly retrospective studies, it found White patients had higher survival to hospital discharge after cardiopulmonary resuscitation than Black patients (OR 1.36, 95% CI 1.22–1.50), and that comorbidity (OR 0.51) and low body mass index (OR 0.64) were associated with lower survival (Du et al.). These are associations, not causal effects. Who receives timely, high-quality resuscitation is itself an implementation question as these disparities imply that fidelity to the intervention is varying across populations.

The three completed implementation evaluations, the nerve block program, the Spanish screening program, and the Tanzanian intervention, share an explicit framework, engaged partners, attended to local context, and had pre planned measurement collections and analysis. The difficulties of implementation are also shared across contexts: define the intervention, tailor it locally, iterate as conditions change, and sustain trust and data systems long enough for any of it to matter.

The Research Topic's clearest limitation is geographic. By World Bank income group, nine contributions come from high-income countries (five from the United States and one each from Sweden, Japan, Poland, and Spain), one from an upper-middle-income country (China), and two from lower-middle-income countries (Tanzania and Nepal); the meta-analysis pools studies from several countries, with all race-stratified data from the United States (5). Upper-middle-income countries fall within the LMIC category, so three contributions are LMIC-based rather than two. No low-income country is represented, both lower-middle-income studies address routine care rather than disaster or outbreak preparedness, and the only LMIC contribution on a climate hazard is the Chinese survey, from an upper-middle-income setting.

Rather than the loose term “LMIC-led,” we apply a criterion readers can check against published author affiliations: whether the first or senior author is based at an institution in the study country. The Chinese survey meets it, with both authors at Hainan Medical University (Wei et al.). Neither lower-middle-income-country study does. The Tanzanian intervention lists first and senior authors at Duke University (Pyne et al.), and the Nepali survey lists both at Southern Medical University in China (Sah et al.), with in-country co-investigators in between. This describes the Research Topic rather than judging the teams, several of which are long-standing partnerships. It matters because disaster mortality falls disproportionately on lower-income countries, which also have the thinnest emergency care systems and health information infrastructure (6, 7).

This Research Topic of implementation literature highlights four priorities in future work. First, treat implementation science as part of routine emergency and disaster leadership, so that tailoring strategies to locally identified barriers and building coordination structures become ordinary rather than exceptional. Second, direct funding and authorship toward investigators based in LMIC and fragile-system institutions, with the authorship criterion above as one verifiable marker. Third, invest in methods fit for emergencies: rapid, pragmatic, and hybrid designs that tolerate evolving evidence and constrained data (8). Fourth, count equity, trust, and resilience as explicit implementation outcomes rather than byproducts.

These contributions show how implementation science can sharpen practice across compound hazards, outbreaks, and everyday emergency care. They also expose blind spots, above all the under-representation of low-income and lower-middle-income settings and of investigators based in them. Emergency and disaster medicine is unlikely to close its evidence-to-practice gap until implementation science becomes central to the field, and until that science reflects the settings it serves.
